# Tissue Preservation Using Socket-Shield Technique in Lower Molar Site: A Proof of Principle Report

**DOI:** 10.3390/dj13040145

**Published:** 2025-03-27

**Authors:** Regimantas Simuntis, Paulius Tušas, Aušra Ražanauskienė, Vygandas Rutkūnas, Marijus Leketas

**Affiliations:** 1 Independent Researcher, 00128 Palanga, Lithuania; rsimuntis@yahoo.com; 2 Institute of Dentistry, Faculty of Medicine, Vilnius University, 01513 Vilnius, Lithuania; vygandas.rutkunas@mf.vu.lt; 3 Independent Researcher, 08339 Vilnius, Lithuania; ausra.razanauskiene@icloud.com; 4 Department of Oral and Maxillofacial Surgery, Lithuanian University of Health Sciences, 44307 Kaunas, Lithuania; marijus.leketas@lsmu.lt

**Keywords:** socket-shield technique, molar area, alveolar ridge preservation, immediate implant placement, buccal plate, dental implantology

## Abstract

**Background/Objectives:** The socket-shield technique (SST) was developed to preserve the facial/buccal portion of a tooth root to prevent post-extraction ridge resorption. It has gained attention for use in anterior implant sites, but its application in posterior sites remains unexplored. The aim of this case report was to report a proof-of-principle case using SST in a lower molar site and evaluate its effectiveness in preserving tissues. **Methods:** A 34-year-old non-smoking patient with a non-restorable mandibular first molar (tooth #36) underwent immediate implant placement with the SST. The tooth’s crown was removed, and the buccal segments of the roots were retained as “shields” while the implant was placed in the center of the socket. Preoperative and postoperative cone-beam CT (CBCT) scans and clinical exams were used to assess outcomes up to 12 months. **Results:** The SST procedure was completed uneventfully. CBCT after 4 months and 12 months showed minimal horizontal bone loss: ~0.2 mm at 4 months; ~0.1 mm additional loss by 12 months. The peri-implant soft tissue profile remained stable, and the implant achieved osseointegration with high primary and secondary stability. **Conclusions:** In this clinical case, the socket-shield technique effectively preserved alveolar bone and soft tissue contours in a molar extraction site, avoiding the ridge collapse often seen post-extraction. This suggests SST may be a viable tissue preservation approach in posterior sites; however, long-term follow-up and further studies are needed to confirm sustained outcomes and validate the technique’s predictability.

## 1. Introduction

In recent years, various approaches have been proposed to preserve the extraction socket and mitigate alveolar ridge resorption, including immediate implant placement, flapless (no-flap) surgery, guided bone regeneration (GBR), and socket sealing techniques [1,2,3]. These methods each confer some benefits, but none of them completely prevent post-extraction buccal bone loss since they fail to retain the periodontal ligament (PDL).

The socket-shield technique (SST) was first described in 2010 as an innovative method to prevent facial alveolar ridge resorption by retaining a portion of the tooth root on the buccal side [4]. The essence of this technique is that the preserved root segment maintains the PDL on the buccal plate, thereby sustaining the blood supply to the buccal bone that would otherwise be lost upon extraction [5]. Since its introduction, the SST has been primarily applied in the anterior aesthetic zone, where maintaining ridge contour is critical [6,7,8,9]. However, alveolar ridge collapse in posterior sites can also lead to significant problems [10,11,12]—not only esthetic deficits but functional issues such as food impaction and plaque accumulation, which may increase the risk of peri-implantitis [13,14]. Despite these concerns, reports of SST in molar regions are scarce; to date, only one isolated case report in a single maxillary first molar case has been published [15].

This article presents a clinical case of the socket-shield technique in a lower first molar site with a 12-month follow-up, highlighting the surgical protocol, short-term results, and considerations in comparison to other techniques.

## 2. Materials and Methods/Technique/Results

**Figure 1:**
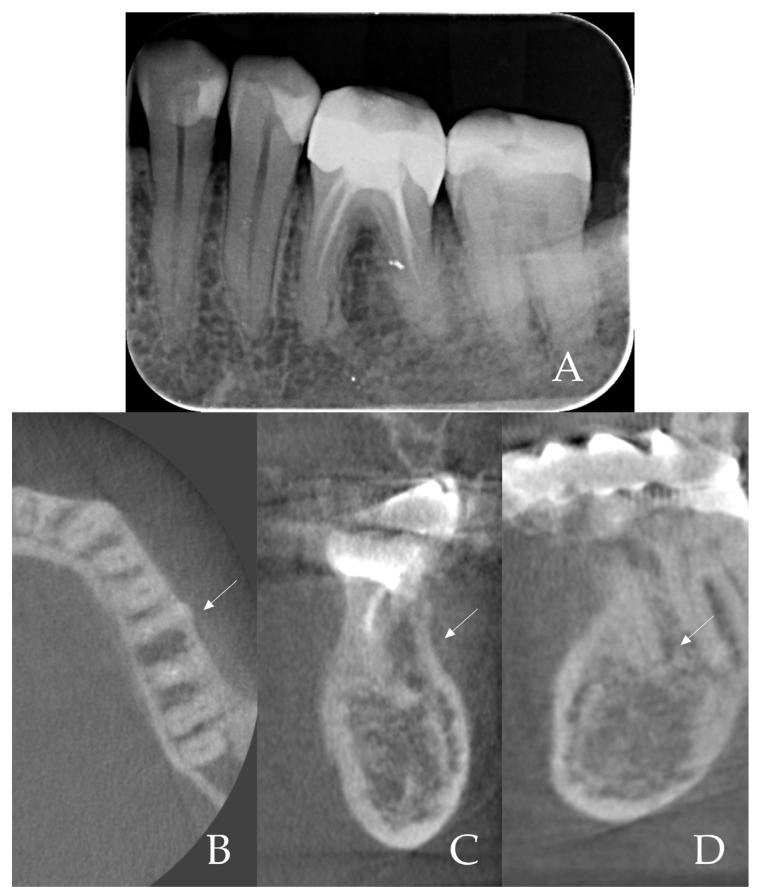
Radiographic preoperative examination. (**A**)—Periapical radiograph of tooth 36, indicating periapical bone destruction around M and D roots; (**B**,**C**)—preoperative CBCT view, indicating an intact buccal cortical plate marked by white arrows; (**D**)—CBCT view, white arrow indicating bone destruction around distal root apex.

Case Presentation: A 34-year-old male patient (non-smoker) presented with a symptomatic, non-restorable mandibular left first molar (tooth #36). The tooth had a large composite filling and had undergone root canal treatment in the past; a crack in the pulp floor extending to the mesiolingual root was detected during a root canal retreatment attempt. The patient refused root canal retreatment and restoration due to a poor prognosis. Periodontal examination showed no active periodontal disease (probing depths up to 3 mm, with no sites of bleeding on probing). A preoperative periapical radiograph and cone-beam CT scan confirmed a periapical radiolucency associated with the mesial and distal roots and an intact buccal cortical plate (Figure 1A–D). Given the poor prognosis of the tooth, an immediate implant placement with the SST was planned to preserve the surrounding bone and soft tissue. The treatment was carried out following the SST guidelines described by Gluckman et al. [15] and in accordance with the principles of the World Medical Association Declaration of Helsinki. Written informed consent was obtained from the patient prior to treatment.

**Figure 2:**
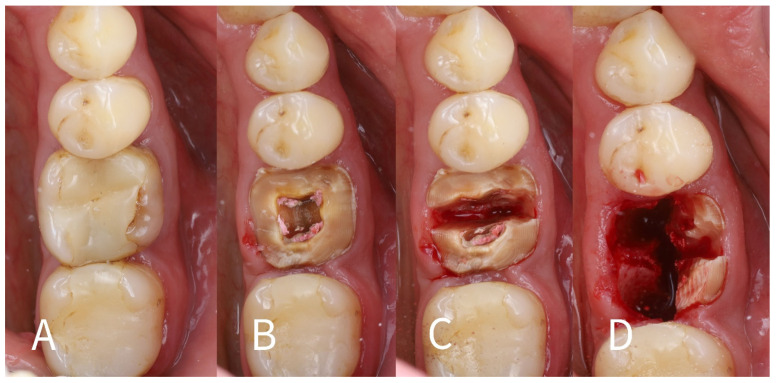
(**A**)—Preoperative intraoral view of the non-restorable mandibular first molar (#36) showing a cracked crown; (**B**)—Decoronation of the tooth; (**C**)—Separation of the mesial and distal roots; (**D**)—Removal of the lingual root fragments (mesial and distal) along with their apices.

**Figure 3:**
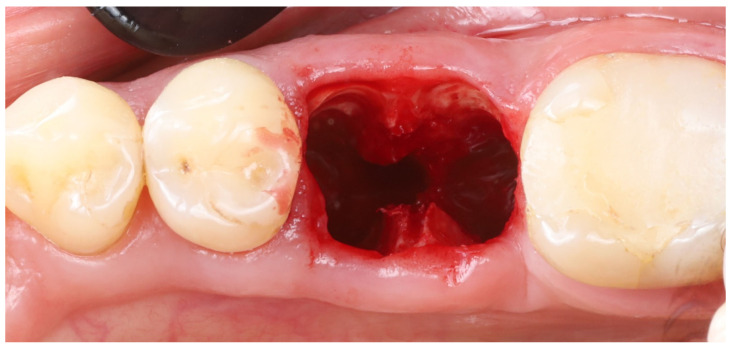
Socket shields after thinning to ~1 mm thickness and trimming flush with the crestal bone level.

**Figure 4:**
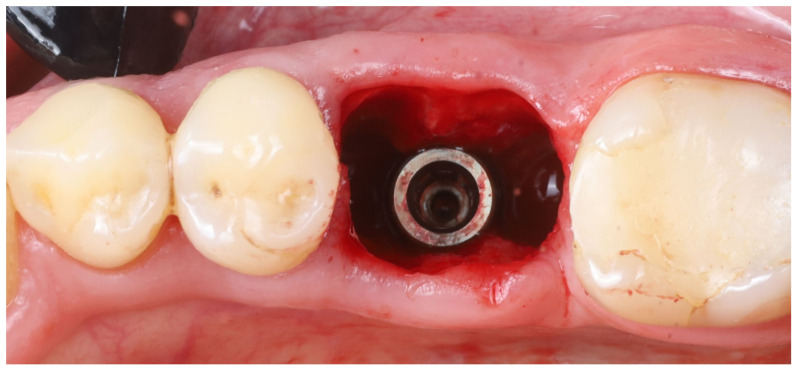
The implant placed ~6 mm apical to the gingival margin, centered in the socket between the two prepared buccal shields.

**Figure 5:**
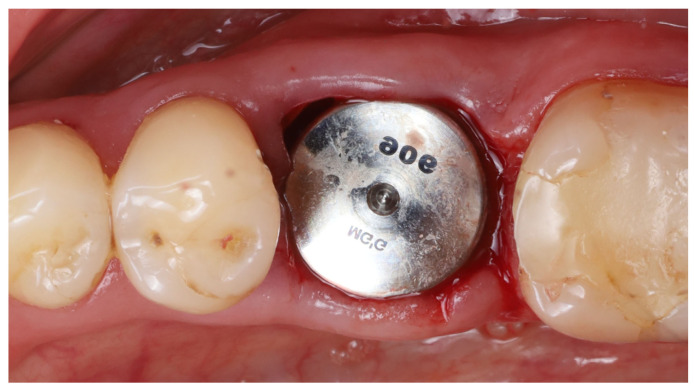
A wide (9 mm diameter) healing abutment used to seal off the socket opening.

**Figure 6:**
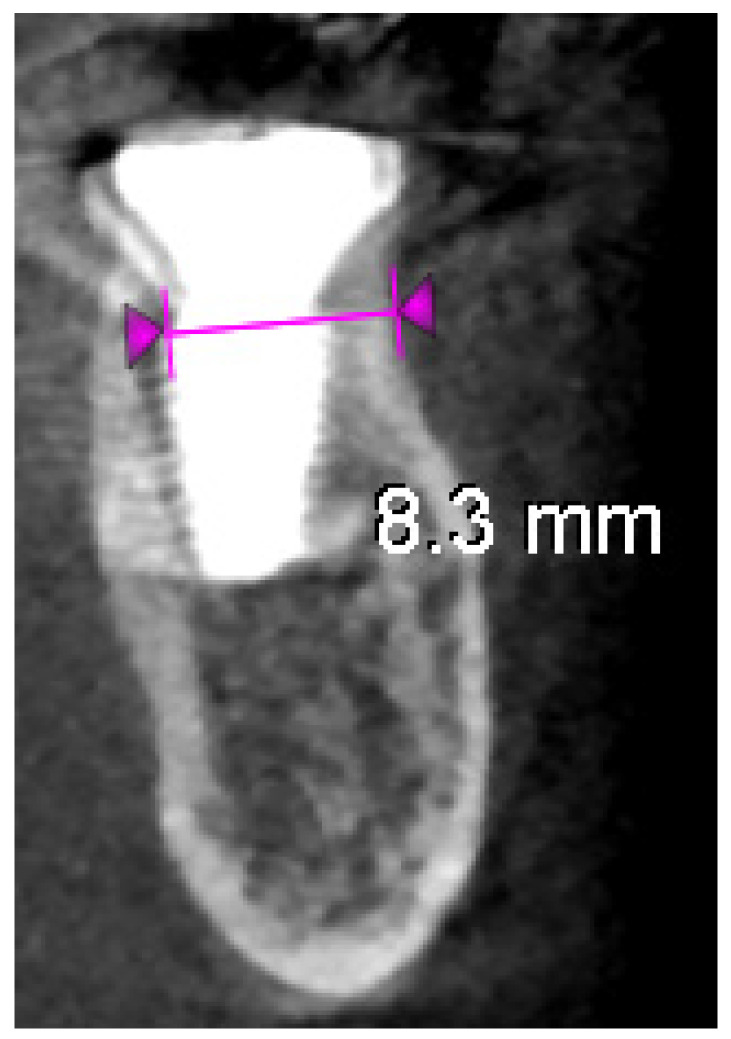
Postoperative CBCT scan showing the implant centrally positioned in the socket.

**Figure 7:**
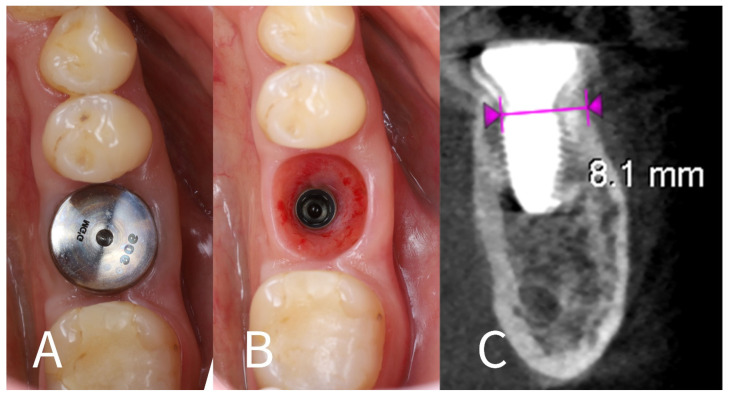
Four-month follow-up. (**A**)—Intraoral view at 4 months post-op, showing that the buccal gingival margin (arrow) remains at the same level as immediately after surgery, while the lingual side shows slight recession; (**B**)—Healthy and well-adapted soft tissues after removal of the healing cap at 4 months; (**C**)—4-month post-op CBCT cross-section, indicating minimal horizontal bone resorption (~0.2 mm) on the buccal aspect (measurements between violet arrowheads) due to the socket shield.

**Figure 8:**
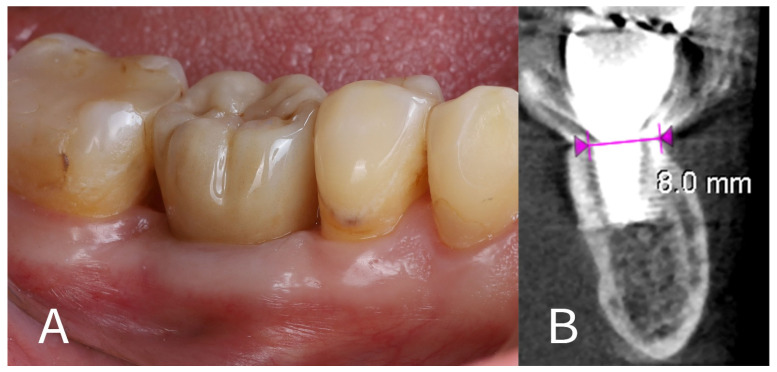
Final outcome. (**A**)—Definitive zirconia crown in place on the implant; (**B**)—One-year post-op CBCT showing maintenance of the buccal bone plate and ridge width, with only ~0.1 mm of additional horizontal resorption on the buccal side (measurement between violet arrowheads) compared to the 4-month image.

Surgical technique: The step-by-step procedure for the immediate implant placement with socket shields was as follows:

1. Root sectioning: After performing mandibular nerve block anesthesia, the crown of tooth #36 (Figure 2A) was removed (decoronation was performed) (Figure 2B). The mesial and distal roots were then separated buccolingually using a long-shanked, straight diamond bur in a high-speed handpiece with copious irrigation (Figure 2C). The preoperative CBCT was consulted to guide the depth and orientation of the cuts, accounting for the roots’ length, curvature, and angulation.
2. Partial root extraction: Each root was sectioned in a mesiodistal direction approximately 1–1.5 mm buccally from the center of each root surface to a depth of about two-thirds of the root length, using a #2 long cylinder bur from Gluckman’s partial extraction therapy (PET) surgical kit (Megagen, Seoul, South Korea). This created buccal and lingual segments for each root. The lingual root segments (along with their apices) were carefully extracted, leaving only the buccal portions of the roots in place as the “socket shields” (Figure 2D). If the lingual part fails to extract and remains in the socket still attached to the shield, it should be detached from the shield with bur #3 and extracted in the conventional way. In such cases, the bony septum can be drilled in the center to facilitate extraction.

3. Shield preparation (thinning): The remaining buccal root segments were refined to serve as socket shields. Each shield was thinned to approximately 1 mm thickness using a large round diamond bur (#3), and the internal (lingual) surface of each shield—facing the future implant—was smoothed with a finer grit bur (#4) (Figure 3). It is important that the shields are thin; leaving them thicker than ~1 mm can increase the risk of complications such as inner (internal) shield exposure during healing.

4. Shield length adjustment: If necessary, the apical extent of each buccal shield was reduced using the same large round bur. The goal was to ensure that each shield’s total height was no more than roughly two-thirds of the original root length. This prevents the shield from extending too far apically beyond where the implant will be placed.
5. Crestal trimming of shields: The coronal height of each shield was reduced to the level of the surrounding buccal alveolar bone crest. This was achieved with finer burs (#5 and #6). Ensuring the shield does not protrude above the bone is critical, as a shield that is too high can become exposed through the gingiva (external shield exposure) [7].
6. Socket debridement: The extraction sockets were thoroughly debrided, especially in the apical areas, to remove any residual soft tissue or infection (granulation tissue). The sockets were then vigorously irrigated with sterile saline to clean out any small root/bone particles created during drilling. This step helps create a clean environment for healing.
7. Intraoperative radiographic check: A periapical radiograph can be taken intraoperatively to verify that no residual root fragments, root canal filling material, or root apices were left behind. This radiographic check can confirm that only the intended buccal root shields remained in situ.
8. Shield stability verification: Each buccal shield was tested for stability by probing gently with a sharp explorer on its internal aspect. Both shields remained completely immobile. (If a shield had exhibited mobility, the socket-shield approach for that root would be aborted, as a mobile or dislodged shield could compromise healing.)
9. Shield integrity check: Using magnification (surgical loupes), the shields were inspected for any cracks or fractures. Both shields were intact, with no visible cracks. If a shield were found to be cracked, it would also necessitate abandonment of the SST for that site because a fractured shield could lead to infection or unpredictable bone healing.
10. Implant osteotomy preparation: An implant osteotomy was created in the inter-radicular septum (furcation bone) of the extraction site [16]. Sequential drilling (using the standard Blue Diamond implant drill kit to the 4.3 mm diameter, Megagen, South Korea) was performed. Care was taken to angle the drills to stay in the middle of the socket and avoid encroaching on the buccal or lingual plates. The osteotomy was prepared to a depth such that the future implant platform would be positioned about 5–6 mm apical to the original buccal gingival margin (as estimated from the CBCT and clinical measurements). This depth was planned to place the implant platform approximately 3–4 mm below the alveolar crest and ~6 mm below the gingival margin once the implant was in place (accounting for the soft tissue height).
11. Implant placement: A dental implant (Megagen Blue Diamond, 4.8 mm diameter × 10 mm length, conical “Deep thread” design) was inserted into the prepared osteotomy. The implant was centered within the socket, with its shoulder positioned roughly 5–6 mm below the level of the surrounding gingival margin (Figure 4). The implant fits snugly between the two buccal shields without exerting undue pressure on them. Primary stability was achieved; the implant had an insertion torque of about 35 N·cm, and verification with a resonance frequency analysis device showed an Implant Stability Quotient (ISQ) exceeding 70. (This high primary stability indicated that the implant was well stabilized in the septal bone despite the immediate placement.)

12. Socket sealing with healing cap: The socket orifice was sealed with a large-diameter healing abutment instead of suturing a flap. A wide, 9 mm diameter, and 6 mm height standard healing cap was attached to the implant, which effectively covered the socket opening and conformed to the circumference of the socket (Figure 5). This approach is intended to protect the socket and support the surrounding gingival margins without requiring a flapped closure or graft. No additional bone graft or membrane was placed in the socket; a blood clot was allowed to fill the gaps between the implant and shields [17].

13. Immediate post-op imaging: A postoperative CBCT scan was obtained to evaluate the implant position and to measure the alveolar ridge dimensions after implant placement (Figure 6). CBCT confirmed that the implant was accurately centered in the socket.

14. Postoperative care: Standard post-surgical care was implemented. The patient was instructed to rinse twice daily with a 0.2% chlorhexidine mouthwash for 2 weeks to aid plaque control during healing. A short course of systemic antibiotics was prescribed (amoxicillin 500 mg, three times a day for 3 days) as prophylaxis, and an NSAID analgesic was advised as needed for pain management. The patient was given routine post-extraction and implant care instructions, with an emphasis on gentle oral hygiene around the surgical site.
15. Follow-up in early healing: The patient was seen for follow-up at 1 week and 2 weeks post-surgery. Healing was uneventful; there were no signs of acute infection or abnormal inflammation. The soft tissue around the healing abutment remained healthy, and no exposure of the shields was observed at the gingival margin.
16. Four-month assessment:

(a) Soft tissue and stability: 4 months after surgery, the site was re-evaluated prior to proceeding with prosthetic restoration. The healing abutment was removed to examine the implant and surrounding tissue. The soft tissues appeared healthy and well-adapted, with a band of keratinized gingiva present. Notably, the gingival margin on the buccal side remained at roughly the same level as immediately after surgery, whereas on the lingual side, a slight recession of the gingiva was noted (this was expected due to the lack of a lingual shield). The implant’s osseointegration was confirmed by a high stability reading (ISQ ~82 at 4 months). There was no pain or mobility, indicating successful initial integration.

(b) CBCT and observations: A CBCT scan at the 4-month mark was taken to quantitatively assess bone healing (Figure 7A–C). The imaging demonstrated that the alveolar ridge width at the implant’s neck had been preserved: horizontal bone loss on the buccal side was measured at only ~0.2 mm compared to the immediate post-op scan. The buccal shield–bone complex was intact and appeared to be maintaining the contour of the ridge. Clinically, the buccal gingiva was in the same position as on the day of surgery (no collapse or loss of buccal tissue height), corroborating the radiographic findings. Figure 7A,B show the soft tissue condition at 4 months: the buccal gingival margin is unchanged, while the lingual gingival margin is slightly lower than before, and the soft tissues are firm and inflammation-free around the healing cap.

17. Prosthetic rehabilitation: At approximately 4 months after the extraction and implant placement, the site was restored with a permanent prosthesis. A screw-retained monolithic full-contour zirconia crown (KATANA Zirconia™ YML, Kuraray Noritake Dental Inc., Tokyo, Japan) was fabricated and attached to the implant (Figure 8A). The crown was designed to have an emergence profile that matched the contour shaped by the healing abutment and surrounding tissue, thereby maintaining the ridge profile.
18. One-year follow-up: The patient was re-examined again at 12 months post-implant placement (roughly 8 months after crown delivery). The patient reported excellent function with the implant and no discomfort. A final CBCT scan at 1 year showed that the alveolar ridge dimensions were unchanged from the 4-month scan (Figure 8B). The horizontal dimension of the buccal bone had an additional resorption of only ~0.1 mm between the 4-month and 12-month time points, indicating stable hard tissue conditions. The buccal plate remained intact and fully supported the implant. The peri-implant soft tissues were healthy, with no signs of recession or inflammation around the crown. The overall result at 1 year was a stable implant with well-preserved surrounding tissues, fulfilling both functional and aesthetic expectations for a posterior implant site.

## 3. Discussion

Tooth extraction initiates a cascade of biological events that lead to alveolar bone resorption, most notably on the buccal side of the ridge [18,19]. The primary cause of this resorptive pattern is the loss of blood supply formerly provided by the periodontal ligament—once the tooth is removed, the buccal bone is nourished only by the limited blood flow from the marrow and the periosteum [4,5]. Consequently, significant ridge width reduction can occur within the first few months post-extraction, potentially compromising implant placement or the aesthetic outcome. In the anterior region, such bone loss can manifest as a visible collapse of the facial gingiva and papillae, undermining aesthetics [10,11]. In posterior regions, where aesthetics is less of a concern, a collapsed ridge can still pose functional problems: it may create a niche for food impaction and plaque accumulation, contributing to patient discomfort and difficulty in hygiene maintenance [14,20]. Over time, these factors can increase the risk of peri-implant mucositis or peri-implantitis in the affected site [14,21]. Therefore, preserving tissue volume after extraction is important not only for aesthetic reasons but also to ensure a functional and cleansable result in implant therapy.

Multiple techniques have been developed to minimize post-extraction ridge resorption. Immediate implant placement is a widely practiced strategy that takes advantage of the existing socket walls to support an implant right after extraction. This approach can reduce the overall treatment time and has some preservation effect by “tenting” the socket, but the remodelling of the bone is not entirely avoided [2]. Performing the implant placement in a flapless manner (i.e., without raising a mucoperiosteal flap) further helps by maintaining the periosteum on the bone, which preserves an extra source of blood supply. Studies have indicated that flapless implant surgery results in less horizontal bone loss compared to cases where a flap is elevated for extraction and implant placement [3]. Another method to counteract ridge reduction is guided bone regeneration at the time of or after implant placement. This typically involves placing bone graft material (autograft, allograft, xenograft, or synthetic) into the socket or around the implant and covering it with a resorbable or non-resorbable membrane. A recent systematic review and meta-analysis found that using bone substitutes in immediate implant sites significantly reduces horizontal buccal bone resorption (on the order of 0.5 mm less loss compared to not using graft) and can improve the peri-implant soft tissue aesthetics [18,22]. However, grafting and GBR often require additional surgeries and costs, and they carry risks such as membrane exposure or graft infection. Moreover, even with grafts, the fundamental biological process of outer bone wall remodelling is only partially mitigated, not completely stopped.

An alternative approach for ridge preservation is the use of socket-sealing techniques. One example is employing a barrier that seals the socket entrance, such as a free soft tissue graft (sometimes called “socket seal surgery” when a palatal tissue graft is used to cover the socket). Another example, which is more relevant in implant cases, is using a customized healing abutment—essentially creating an immediate interim prosthesis or healing cap that fills the socket space and supports the gingival tissues from collapsing inward [1,12]. Ruales-Carrera et al. (2019) described managing immediate implant sites with tailored healing abutments to shape the emergence profile and reported favourable peri-implant tissue outcomes [1]. These “socket seal abutments” can maintain the soft tissue contour and encourage soft tissue healing in the shape of the original tooth, thus preserving the ridge profile during the healing phase. While such methods are effective for soft tissue preservation and can reduce the need for extensive grafts, they do not prevent the internal bundle bone resorption that occurs due to loss of the PDL. In other words, they help maintain the gingival shape but rely on natural bone healing or grafting beneath—they do not actively halt the bone resorptive process.

The socket-shield technique, in contrast, is a biologically oriented method that directly addresses the cause of buccal bone loss by preserving the tooth’s PDL on the buccal aspect [4,5]. By leaving the buccal segment of the root in place, the blood vessels in the PDL and the bundle bone of the buccal plate remain largely intact, which can effectively prevent the usual post-extraction ischemia and bone remodelling. This innovative approach was first demonstrated by Hürzeler and colleagues in 2010 with histologic evidence that new bone can form behind a retained root fragment and alongside an implant without adverse effects [4]. Since then, several reports and small case series have documented successful outcomes using SST, primarily in the anterior maxilla [6,8]. The evidence, while still evolving, indicates that SST can significantly minimize buccal bone resorption when compared to conventional extraction and grafting approaches. In a recent systematic review of longitudinal studies (including randomized trials and cohort studies), Kotsakis et al. found that retaining the buccal root segment in immediate implant placement led to markedly less marginal bone loss and buccal plate change, while implant survival and success were comparable to the traditional approach [2]. Similarly, a randomized clinical trial by Abd-Elrahman et al. in 2020 reported that the socket-shield technique (with immediate temporization) resulted in significantly reduced labial bone loss and superior gingival tissue levels compared to immediate implant placement with complete extraction [23]. These studies validate the concept that preserving the buccal root segment is beneficial for hard and soft tissue stability.

Schwimer et al. in 2019 published a proof of principle case report on the maxillary first molar by leaving the shields of the mesiobuccal and distobuccal roots with encouraging results after 4 months. In that case, a flap was raised to visualize the buccal wall, gaps filled with bone particles, and the socket was sealed with a small diameter (5 mm) gingival cap and sutured, gaps left for secondary healing [15]. In this case, a flap was not raised to preserve the blood supply to the buccal plate. Also, it was decided to leave the gaps for the blood clot to fill; therefore, no additional foreign materials could interfere with natural bone formation between implant and shield. Finally, the socket was sealed with a large diameter gingival cap, which conforms ideally to the socket circumference, which accelerated the healing process. The most common complication that may occur during this procedure is internal and external shield exposure [20], which is caused by excessive height and width of the shield. In this case, the shield was trimmed to the bone level and thinned to about 1 mm to avoid these complications.

The 12-month results we achieved align with the favourable outcomes reported in the literature. By clinical and radiographic measures, our patient’s buccal bone and gum contour were preserved: only ~0.1–0.2 mm of buccal bone remodelling occurred, and the buccal gingival margin remained at almost the same position as on the day of surgery. For illustration, Figure 7; Figure 8 provide a comparison of the buccal view before treatment and at the 1-year follow-up, demonstrating virtually no collapse of the ridge profile. This stability is in stark contrast to the ridge resorption often seen in molar sites treated with extraction and delayed implant placement (or even with some immediate placements without SST). Functionally, the preserved contour has likely helped the patient avoid food trapping in the area and made hygiene around the implant easier, as there is no significant horizontal deficit. However, it is important to note, that we intentionally did not use any adjunctive bone graft in this case in order to examine the sole effect of the socket shield and blood clot healing; in cases with large gaps or defects, a combined approach (SST + biomaterials) might be considered.

Despite the promising outcome observed, this case report has inherent limitations and highlights points of caution. Firstly, the socket-shield technique is highly technique-sensitive. In molar sites especially, the clinician must carefully section and remove the correct portions of the roots without damaging the buccal plate or dislodging the buccal fragment. The presence of multiple roots and inter-radicular septal bone can make shield preparation more complex than in single-rooted teeth [9,15]. Only clinicians experienced in immediate implant techniques, and PET should attempt the socket-shield technique in such scenarios. In our case, meticulous attention was given to thinning and shortening the shields; this likely contributed to the uneventful healing, as excessively thick or tall shields are known to risk exposure [7]. Secondly, the follow-up period of this report is relatively short (12 months). While the results at 1 year are encouraging, the long-term behaviour of the socket-shield interface and the surrounding bone is still not fully understood. Thirdly, as a single-case report, our findings serve as a proof of principle rather than a generalizable outcome. No control comparison was made in this report (e.g., with a conventional technique in the same patient), and individual patient factors could influence the result. While SST clearly preserved the tissues in this instance, its success can be case-dependent.

Within these limitations, this case adds to the growing evidence that the socket-shield technique can be an effective means of tissue preservation in implant dentistry. It demonstrates that even in a molar site—traditionally considered prone to resorption—the careful retention of buccal root segments can preserve the ridge and simplify subsequent restoration. Future research, including well-designed controlled clinical trials and long-term cohort studies, is necessary to further validate the SST.

## 4. Conclusions

Within the limits of a single-case observation, this report highlights the potential benefits of the socket-shield technique for preserving alveolar ridge integrity in a posterior site. The SST in a lower molar region allowed immediate implant placement while largely preventing the buccal bone and soft tissue loss that typically follows extraction. One year post-treatment, the implant showed stable osseointegration, and the peri-implant tissues remained healthy with minimal dimensional change. These findings suggest that SST can be a valuable technique to improve functional and aesthetic outcomes of implants, especially in situations where maintaining ridge contour is challenging. Successful application of the socket-shield technique requires careful case selection and surgical precision, as mistakes in shield preparation can lead to complications. Long-term follow-up is still needed to ensure that the initial advantages are maintained over time. Overall, the socket-shield technique may serve as a useful alternative or adjunct to traditional ridge preservation methods (such as GBR, flapless surgery, or socket seal approaches) by directly preserving the patient’s own buccal plate and periodontal ligament. Continued research and clinical reporting will help establish standardized guidelines and determine the predictability of SST in diverse clinical scenarios.

## Data Availability

The raw data supporting the conclusions of this article will be made available by the authors upon request.

## Abbreviations

The following abbreviations are used in this manuscript:

CBCT	Cone-beam computed tomography
GBR	Guided bone regeneration
PDL	Periodontal ligament
PET	Partial extraction therapy
SST	Socket-shield technique
